# Anticipatory Posturing of the Vocal Tract Reveals Dissociation of Speech Movement Plans from Linguistic Units

**DOI:** 10.1371/journal.pone.0146813

**Published:** 2016-01-13

**Authors:** Sam Tilsen, Pascal Spincemaille, Bo Xu, Peter Doerschuk, Wen-Ming Luh, Elana Feldman, Yi Wang

**Affiliations:** 1 Linguistics, Cornell University, 203 Morrill Hall, Ithaca, NY, 14853, United States of America; 2 Radiology, Cornell Weill Medical College, 416 East 55th St. Floor B1, New York, NY, 10022, United States of America; 3 Biomedical Engineering, Cornell University, 135 Weill Hall, Ithaca, NY, 15843, United States of America; 4 Human Ecology, Cornell University, G340A Martha Van Rensselaer, Ithaca, NY, 14853, United States of America; Sun Yat-sen University, CHINA

## Abstract

Models of speech production typically assume that control over the timing of speech movements is governed by the selection of higher-level linguistic units, such as segments or syllables. This study used real-time magnetic resonance imaging of the vocal tract to investigate the anticipatory movements speakers make prior to producing a vocal response. Two factors were varied: preparation (whether or not speakers had foreknowledge of the target response) and pre-response constraint (whether or not speakers were required to maintain a specific vocal tract posture prior to the response). In prepared responses, many speakers were observed to produce pre-response anticipatory movements with a variety of articulators, showing that that speech movements can be readily dissociated from higher-level linguistic units. Substantial variation was observed across speakers with regard to the articulators used for anticipatory posturing and the contexts in which anticipatory movements occurred. The findings of this study have important consequences for models of speech production and for our understanding of the normal range of variation in anticipatory speech behaviors.

## Introduction

When we speak, we control the selection and execution of numerous articulatory movements. Current models of speech production assume that movement initiation is organized by higher-level linguistic units, such as segments and/or syllables [1–3]. However, recent studies suggest that speech movements associated with the same segment or syllable can be dissociated from each other [4,5]. This phenomenon may be particularly prevalent immediately prior to the beginnings of utterances, when speakers can anticipate upcoming motor plans and are maintaining those plans in working memory. Yet little is currently known about the contexts in which pre-speech anticipatory movements can occur, as well as the vocal organs that may be involved and the range of interspeaker variation. Moreover, studying anticipatory behavior before utterance initiation may inform our understanding of coarticulatory patterns in running speech.

**Fig 1:**
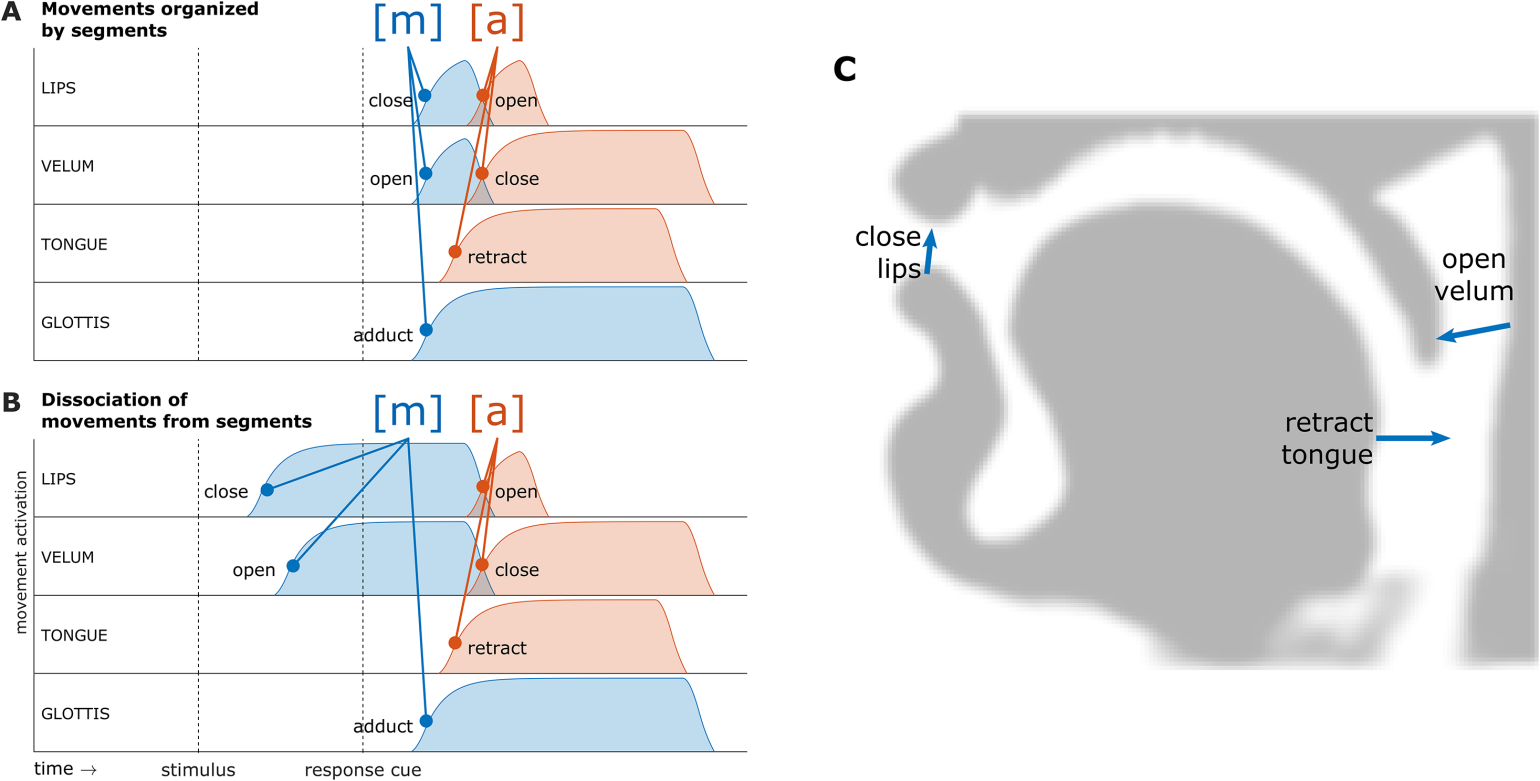
Schematic comparison of predicted articulatory movements in the syllable [ma]. (A) Movement initiation is organized by segments. (B) Dissociation of movements from linguistic units: anticipatory closure of the lips and opening of the velum well in advance of the response. (C) Mid-sagittal vocal tract profile illustrating movements involved in the production of [m].

To illustrate the main theoretical issue, Fig 1 schematizes the predictions of two generic models of articulatory control in the syllable "*ma*", produced in a delayed response task. To produce an [m], a speaker closes their lips, lowers their velum lowers to allow nasal airflow, and adducts their vocal folds to phonate. To produce the vowel [a], a speaker opens their lips, retracts their tongue, and raises their velum. The oral movements involved are illustrated in a mid-sagittal profile of the vocal tract in Fig 1C. Of particular importance here is when the movements occur in time relative to each other.

It is commonly assumed that articulatory movements occur in a stereotyped, pre-programmed pattern. Experimental evidence in general accords with this view. For example, the retraction of the tongue for a low vowel like [a] is typically tightly synchronized with the bilabial closure for an [m] or [p] and precedes the release of the bilabial closure [6–8]. This well-replicated finding suggests that the consonantal and vocalic movements are coordinated by a higher-level linguistic unit such as a syllable. For current purposes it is not crucial to distinguish between segmentally and syllabically organized control models because both make similar predictions regarding anticipatory behaviors. Specifically, models which assume movements are organized by segments and/or syllables predict that speakers will not produce any response-specific movements in a delay interval between a stimulus and response cue, as shown in Fig 1A. This prediction follows from the assumption that speakers select linguistic units and initiate their associated movements in a stereotyped or coordinated manner, along with the assumption that speakers wait for a response cue before starting the initiation process. Such models also imply that the relative timing of movements associated with a given segment or syllable should be fairly consistent.

In contrast to segmentally/syllabically organized control models, Fig 1B shows predictions of a model in which the initiations of movements associated with the same linguistic unit can be dissociated from each other. Here the speaker closes the lips and lowers the velum for [m] without adducting the vocal folds or retracting the tongue for the vowel [a]. In other words, the movements associated with the segment [m] or the syllable [ma] need not be initiated closely together in time in a precisely controlled pattern. That such anticipatory behaviors occur seems intuitively obvious, so it is problematic that current models have not described mechanisms for their occurrence.

Pre-speech anticipatory posturing is distinct from anticipatory coarticulation, but likely can inform our understanding of coarticulatory phenomena. Anticipatory coarticulation refers to the phenomenon in which an upcoming articulatory movement or target influences a preceding one. Coarticulation is to some extent a planned aspect of speech [9–11], and substantial variation in the spatio-temporal extent of coarticulation has been observed, along with cross-linguistic, inter-speaker, and contextual variation [12–15]. The multifaceted nature of coarticulation speaks against any monolithic account of the phenomenon. Pre-speech anticipatory movements are not quite the same as coarticulation because they occur during a period of time in which no competing movement targets actively control the posture of the vocal tract; in contrast, anticipatory coarticulation specifically involves an interaction between two or more targets. However, pre-speech anticipatory posturing may be an extremal form of anticipatory coarticulation that occurs in the absence of a competing postural target, and thus may provide insight into the nature of coarticulation.

### Segmentally/syllabically organized models

Current models of speech production assume that movements are organized by linguistic units such as segments, syllables, or even words. Few production models are sufficiently explicit and comprehensive to make predictions about the relative timing of speech movements; here we focus on the Gradient Ordering, Directions into Articulator Velocities (GODIVA) model of [1], the Task Dynamics model of Articulatory Phonology [6,16], and the Action-Based Neurocomputational Model of Speech Production (ACT), [2,17]. The predictions of various other models cannot be evaluated because these models do not explicate relations between linguistic structural organization and motor control processes that govern movement timing (e.g [18,19], see 3).

In the GODIVA model [1], segments organize speech movements and segments are competitively selected to fill syllable positions, which may in turn influence parameters relating to the control of articulation. Because articulatory movements are organized by a combination of their segmental affiliation and syllable position, movements cannot be initiated until their corresponding segment has been selected and associated to a syllable position. There is no mechanism in the model for one movement associated with a given segment to be executed independently of others or to be executed without first being incorporated into a syllabic structure. Once segments are incorporated into the syllabic structure, the relevant control parameters are assumed to be fairly stereotyped.

In the Task Dynamics model of Articulatory Phonology (AP) [6,16] movements are associated with articulatory tasks (termed *gestures*), and the relative timing of gestures is controlled by a "gestural score". Descriptions of the model have varied regarding whether gestures are organized by syllables or words, and whether the timing patterns in gestural scores are lexically stored [16] or derived in the course of speech planning [20]. Notably, more recent versions of the spoken word production model of Levelt and colleagues [21,22] have cited the Articulatory Phonology model as a viable approach to controlling articulatory movements that are organized in syllables. ACT is a hybrid model which incorporates the sensorimotor control mechanisms of GODIVA but like AP views gestures as the fundamental motor units and specifies speech plans with gestural scores [2].

No explicit mechanisms have been incorporated into AP or ACT to allow for gestures to be produced independently of a higher-level organization, but the models do not explicitly rule out the possibility that other processes might allow for speech movements to be dissociated from linguistic units. The assumption that gestures are linguistically organized is nonetheless often made in interpreting experimental results. For example, a study of syllable structure effects on response latency in delayed naming [23] explicitly assumed the organization of gestures into syllabic units. Likewise, a study of gestural intrusion errors in a repetition task in [5] showed that gestures have the ability to dissociate from the syllables they are normally produced in, but the intruding gestures were interpreted as reassociating through coordinative mechanisms with a different linguistically organized set of gestures.

One previously proposed biomechanical mechanism which could be extended to model pre-speech anticipatory posturing involves the neutral vocal tract attractors in the Task-Dynamic model [16]. The neutral attractors were proposed to drive articulator movements when there is no active control. If instead of being “neutral” these vocal tract targets are allowed to vary as a function of upcoming gestures then linguistically dissociated movements prior to initiation of an utterance would be possible. Indeed, vocal tract postures during pauses in speech have been shown to maximize mechanical advantage [24], which means that postures adopted during pauses appear to facilitate rapid changes of articulatory positions relative to changes in vocal tract task goals. Although [24] did not examine response-specific posturing, it is possible that a biomechanical optimization mechanism can drive vocal organs to postures which optimize mechanical advantage in a context-specific manner. This mechanism could operate in parallel with linguistic organization of movement and give rise to pre-speech movement dissociations.

### Dissociability of movements

Various forms of evidence from experimental studies suggest that speech movements can be dissociated from higher-level linguistic units such as segments or syllables. In disyllable repetition tasks designed to elicit speech errors, individual movements can intrude in syllables in which they are not expected [5], and in tongue twister paradigms speakers produce gradient, subsegmental errors in voicing and other features [25]. In tasks probing the interruptibility of speech, speakers can interrupt themselves between movements associated with the same segment [4]. Studies of discrepancies between acoustic and articulatory measurements of verbal reaction time [26,27] have provided suggestive evidence for movement dissociations, and investigations of language-specific "speech-ready" postures [24,28,29] demonstrate that speakers control vocal tract posture prior to speech responses. However, these studies do not specifically distinguish between response-specific anticipatory movements and generic speech-ready postures; a more systematic investigation of response-specific anticipatory movement is therefore warranted.

The current study asked whether individual articulatory movements can be dissociated from segments/syllables in anticipation of an upcoming response, and whether the presence of an active postural target influences this phenomenon. In addition, there are open questions regarding which articulators are involved in such dissociations, and how variable such patterns are across speakers. Data were collected with real-time magnetic resonance imaging (rtMRI), which provides a combination of temporal resolution and spatial resolution/coverage not possible with other technologies [30,31]. Twenty-five adult native speakers of English performed a syllable production task in an MRI scanner, with mid-sagittal vocal tract slices imaged at approximately 200 Hz. Four syllables {*pa*, *ma*, *ta*, *na*} were produced in four blocked conditions corresponding to a 2×2 cross of the factors *response preparation* and *pre-response postural constraint*. Preparation was manipulated by controlling whether participants had knowledge of the response target prior to the response cue. Pre-response constraint was manipulated by requiring participants to produce a prolonged vowel "ee…" prior to the initiation of the response. The following hypotheses were tested:

Hyp. 1: *Dissociation of articulatory movements in anticipation of utterance initiation*. Prior to the initiation of an utterance, the execution of individual articulatory movements can be dissociated from the linguistic units which typically organize them.

Prediction: pre-response vocal tract postures will be more similar to response target postures in the prepared response condition than in the unprepared condition.

Hyp. 2. *Absence of postural constraints facilitates movement dissociations*. There is a greater propensity for dissociations of articulatory movements from linguistic units when no preceding articulatory targets are present.

Prediction: anticipatory posturing effects will be more prevalent in the unconstrained condition compared to the constrained condition.

The predictions of Hyp. 1 follow from the fact that foreknowledge of the articulatory content of the upcoming response is only available in the prepared condition, along with the assumption that speakers can incorporate this knowledge in anticipatory control. If individual movements cannot be dissociated from linguistic units, no differences should between prepared and unprepared responses will be observed. The predictions of Hyp. 2 follow from the observation that the constrained condition mimics the situation of anticipatory coarticulation: a competing target (the posture for “ee”) is active during the period of time in which upcoming articulatory targets might induce anticipatory movements.

## Materials and Methods

### Participants

A total of 25 adult speakers (ages 18–25, 13 female, 12 male) with no self-reported speech or hearing disorders participated in this study. All participants were native speakers of English, but the demographics of the available subject pool precluded control over other details of language background (e.g. other languages spoken, language instruction history). Data from one participant were excluded due to the presence of noise in the MRI images. Written informed consent was obtained from all participants. The study protocol was approved by the Institutional Review Board of Cornell University.

### Tasks and Procedures

Prior to entering the scanner, each participant performed a set of four practice blocks with an experimenter present. The practice blocks corresponded to the four different response conditions in the experiment. The participants were instructed that on each trial of the experiment one of four letters: "p", "m", "t", or "n", would appear on the screen, and that their response would be "pa", "ma", "ta", or "na", accordingly. In addition they were instructed that they should always wait to respond until a large green box appeared on the screen (i.e. a go-signal). Before each practice block participants were read block-specific instructions described below. At the start of each trial a yellow box appeared to signal the speaker to get ready. After a delay of random duration between 1250 and 1750 ms, the green go-signal appeared, signaling the speaker to produce the response. The random delay interval introduces uncertainty in the timing of the go-signal in order to discourage participants from guessing when the signal will appear; on the basis of pilot investigations, the range 1250–1750 ms was deemed large enough to prevent such behavior without unduly lengthening the duration of the experiment. The ready- and go-signals were rectangles occupying 85% of screen width and height.

**Fig 2:**
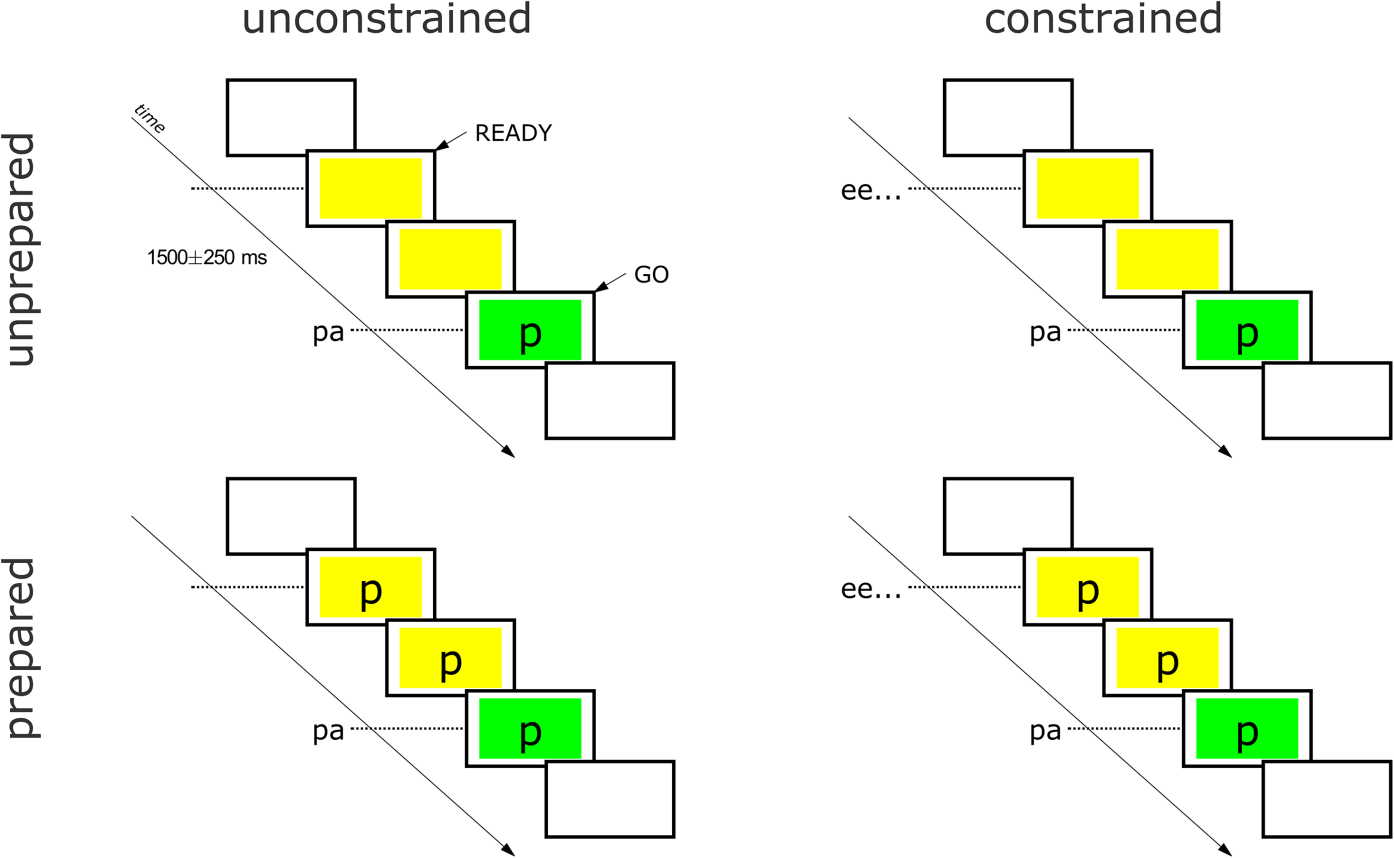
Task design. Blocks of 16 trials were performed with a 2 x 2 blocked design crossing response preparation and pre-response constraint. In constrained blocks, participants produce a prolonged "ee…" in response to the ready signal.

Experiment blocks were comprised of 16 trials, with 4 repetitions of each of the 4 responses elicited in random order. As illustrated in Fig 2, each block can be characterized by two design variables: *pre-response constraint*, i.e. whether or not the speaker produces the high front vowel [i] ("ee…") during the ready phase, and *preparation*, i.e. whether the speaker is given foreknowledge of the response. The preparation conditions were implemented by controlling by the presence of the cue stimulus during the ready phase. The constraint conditions were implemented by block-specific instructions. For the constrained blocks, participants were instructed to say "ee…" when the yellow box appears and to hold "ee…" all the way until producing the response. For the unconstrained blocks participants were instructed not to say anything before the response.

The choices of response targets {pa, ma, ta, na} and pre-response posture “ee” are motivated as follows. The vowel “ee” was chosen for the pre-response constraint because the tongue root movement from “ee” to “aa” (the vowel in all target syllables) is of relatively large magnitude, facilitating robust identification of this movement. Both nasal and non-nasal consonants were included in the response targets to allow for analyses of anticipatory velum posturing. Nonword responses were elicited in order to avoid confounding effects of lexical identity, and responses were monosyllabic rather than multisyllabic in order to avoid potential effects of any articulations subsequent to the response-initial ones.

During the practice phase, participants performed each of the four blocks once in the following order: constrained-unprepared, unconstrained-unprepared, constrained-prepared, unconstrained-prepared. This same order was maintained in the experiment for all participants, because counter-balancing the 24 permutations of blocks across participants or randomizing orders within participants would result in potential speaker-order confounds. Switching tasks between responding immediately (unprepared condition) and waiting (prepared condition) can result in anticipatory errors: the preparation conditions were grouped in the four-block sequence in order to reduce such errors. Each participant performed 6 or 7 iterations of the four-block sequence (depending on time constraints), resulting in a total of 24 or 28 blocks (384 or 448 trials) for each participant. The entire experiment took approximately 90 min. (60 min. for the experimental task, 30 min. for setup/take-down). Participants were also given general instructions to begin the response as soon as possible when the green box appears, to try hard to produce the correct response on every trial, and to try to say the response the same way throughout the experiment (in order to discourage intentionally introduced variation in responses).

During the experiment participants lay supine in the MRI scanner and wore earplugs. A computer monitor behind the bore was visible through a mirror on the head-mounted receiver coil. Each block of trials began 5 seconds after the initiation of the MRI pulse sequence and lasted for approximately 125 seconds. Speech audio was recorded by an optical microphone mounted on the receiver coil approximately 5–10 cm from the speaker's mouth; a reference audio signal was recorded by an identical microphone in the same location oriented in the opposite direction. Audio signals are not analyzed in this study because the research questions can be more directly addressed by examining vocal tract postures in the MRI images.

### MRI data acquisition and image reconstruction

MRI signals were acquired with a 12-channel head-mounted receiver coil in a 3.0T GE Discovery MR750. Variable density golden angle ordered spirals were used in a 2D gradient spoiled echo pulse sequence for data sampling. The following scan parameters were used: FA 5°, FOV 26 cm, BW ±125kHz, acquired matrix size 128 x 128, spatial resolution 2.03×2.03 mm^2^, 24–36 spiral leaves, sagittal slice thickness 20/25 mm, TR 4.072–6.742 ms, TE 0.632–1.554 ms. The acquired slice was approximately midsagittal. Because of scanner firmware changes and efforts to optimize signal quality, scan parameters varied slightly across participants (S1 Table).

Images were reconstructed using the TRACER algorithm [31], which aims to minimize the temporal footprint of each frame while maintaining image quality by imposing consistency between adjacent frames. 10 of the 12 channels were used for image reconstruction; the two excluded channels contained signal from regions outside the vocal tract. The TRACER dynamic tolerance parameter, which limits the change between successive frames by limiting iterations, was optimized for each session by varying the parameter until the average number of iterations per frame over the first 1000 frames of the initial scan in the session was between 2 and 2.5.

### Image processing and feature extraction

Subjects' head movement was minimal due to padding used in the MRI subject bed along with explicit instructions not to move their head. Changes in head position across a session, as estimated in our co-registration process, were typically less than ±3 mm and ±2° in the midsagittal plane. To compensate for head position changes, reconstructed images were co-registered within sessions by applying a rigid transformation. To estimate an optimal rigid transformation the following procedure was employed. For each trial, a trial-mean image was calculated as the mean pixel intensity during a 500 ms period before the go-signal. The reference image for all transformations was taken from the first trial in the block midway through each session. A non-dynamic region of the reference image was defined by locating the vertical pixel index 15 mm above the apex of the palate and masking the reference image below this line; hence the reference image for calculating transformations encompassed solely cranium and cortex. A Euclidean-distance metric based on pixel intensities was used to determine the optimum alignment of each trial-mean image to the reference image. Images were subsequently cropped to a region of interest with 15 mm margins around a rectangle whose sides were defined by the apex of the palate, posterior wall of the pharynx, inferior extremum of the jaw, and anterior extremum of the lips/jaw. The cropped images were upsampled by a factor of 3 using cubic interpolation.

**Fig 3:**
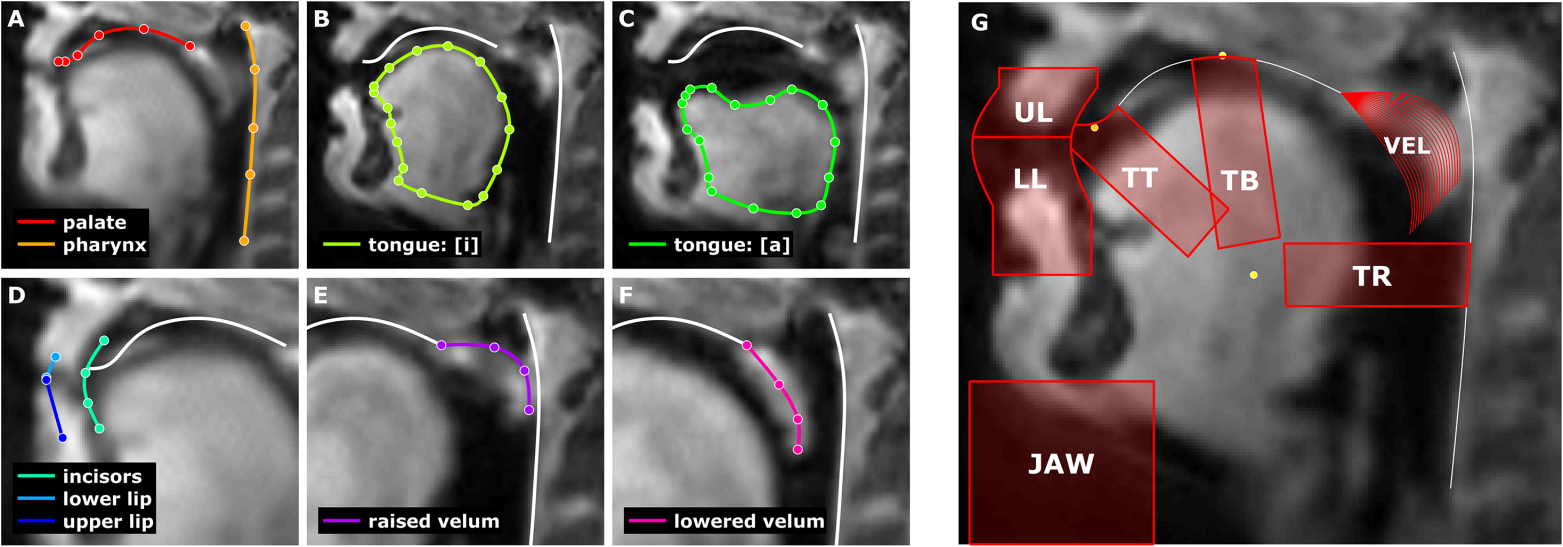
Example of manual labeling of anatomical landmarks and automatically generated regions of interest. Acronyms in panel G: UL (upper lip), LL (lower lip), TT (tongue tip), TB (tongue body), TR (tongue root), VEL (velum).

Anatomical landmarks and contours were estimated for each session by manual landmarking based upon visual inspection of the registered image sequence, using the block midway through the session. Fig 3 panels A-F illustrate the manually determined landmarks: (A) the posterior wall of the pharynx and surface of the palate from velum to upper incisors; (B & C) tongue profile during [i] and [a]; (D) approximate location of dentition line, point of contact of the lower and upper lip during [p]/[m], and extents of upper and lower lips; (E & F) maximally raised and maximally lowered velum. Anatomical contours were estimated by fitting a 2D smoothing spline to the manually located points. Because the hard palate and dentition produce very little signal due to their molecular compositions, these structures are estimated from inspection of the range of articulator movement in image sequences.

### Defining regions of interest and velum structural models

Articulatorily relevant ROIs (Fig 3G) were defined automatically from the manually estimated anatomical landmarks and contours. The upper lip ROI (UL) extends 15 mm superior to and 10 mm anterior to the lip contact point, parallel to the estimated dentition line. The lower lip ROI (LL) extends 30 mm inferior and 10 mm anterior to the lip contact point, parallel to the dentition line. All tongue-based ROIs had a width of 10 mm and were centered on an axis originating from the tongue centroid, defined as the mean of the centroids of the [i] and [a] contours. The width of 10 mm was chosen heuristically: much larger widths conflate movements of different portions of the tongue, and smaller widths result in noisier estimates of articulatory features. The other points defining the ROI axes were the alveolar contact point for the tongue tip ROI (TT), the apex of the palate for the tongue body ROI (TB), and the horizontal projection of the centroid to the pharyngeal wall for the tongue root ROI (TR). In order to diminish overlap between tongue ROIs, these ROIs were truncated along the axis from the centroid by a factor of 1/2 of the distance between the average tongue centroid and [a] centroid, or a factor of 1 in the case of the TT ROI. The ROIs were adjusted to conform to the dentition line, palate, and pharyngeal wall contours. JAW ROIs were defined as a rectangular region from the most anterior and inferior point of the [a] tongue contour to the most anterior point of the UL/LL ROIs and the bottom of the image.

Because the motion of the velum does not lend well to ROI-based analyses, an alternative approach was employed using correlation with structural models. For each session, twenty structural models of the velum were defined by interpolating contours between the maximally lowered and maximally raised contours. Each interpolated contour was converted to a structural model by two single-pixel dilations of the contour, enforcing a linear decrease from 1 to 0.33 in pixel intensity for each successive dilation.

### Articulatory feature extraction

Registered images were contrast-enhanced using an anisotropic diffusion algorithm [32] with integration constant 1/7, gradient modulus threshold 10, 25 iterations, and the wide-region conduction coefficient function. Subsequently articulatory features were extracted from the enhanced images using the articulatory ROIs and velum structural models. To extract articulatory features representing the positions of the upper lip, lower lip, tongue tip, tongue body, and tongue root, the pixel intensity centroid (ROI-IC)—i.e. an intensity-weighted average spatial position—was calculated for each of the corresponding ROIs on a frame-by-frame basis. The ROI-IC represents the mean spatial location of tissue in an ROI, analogous to the center of gravity of an arbitrary two-dimensional object with varying density. We note that the ROI-IC is distinct from previous ROI-based analyses which use mean ROI pixel intensity or pixel intensity correlations [33,34]: intensity centroids are spatial positions, as opposed to aggregate measures of pixel intensity. Although the ROI-IC feature does not represent the location of an edge or anatomical structure, it does reflect the direction of tissue movement into and out of an ROI as well as the positions of articulators within an ROI. For the velum feature, the mean pixel-wise product between each structural model and the image was calculated, and the index of the maximally correlated model was chosen as the feature value. All articulatory feature time-series were resampled to 500 Hz, and fit with a cubic smoothing spline (smoothing parameter 0.9995). A lip aperture (LA) feature was defined as the Euclidean distance between the UL and LL ROI-ICs. Because the tongue tip movement for coronal consonants can involve both anterior-posterior and inferior-superior movement, the first principle component of the TT time series was calculated for each trial using a 4s window centered on the go-signal.

**Fig 4:**
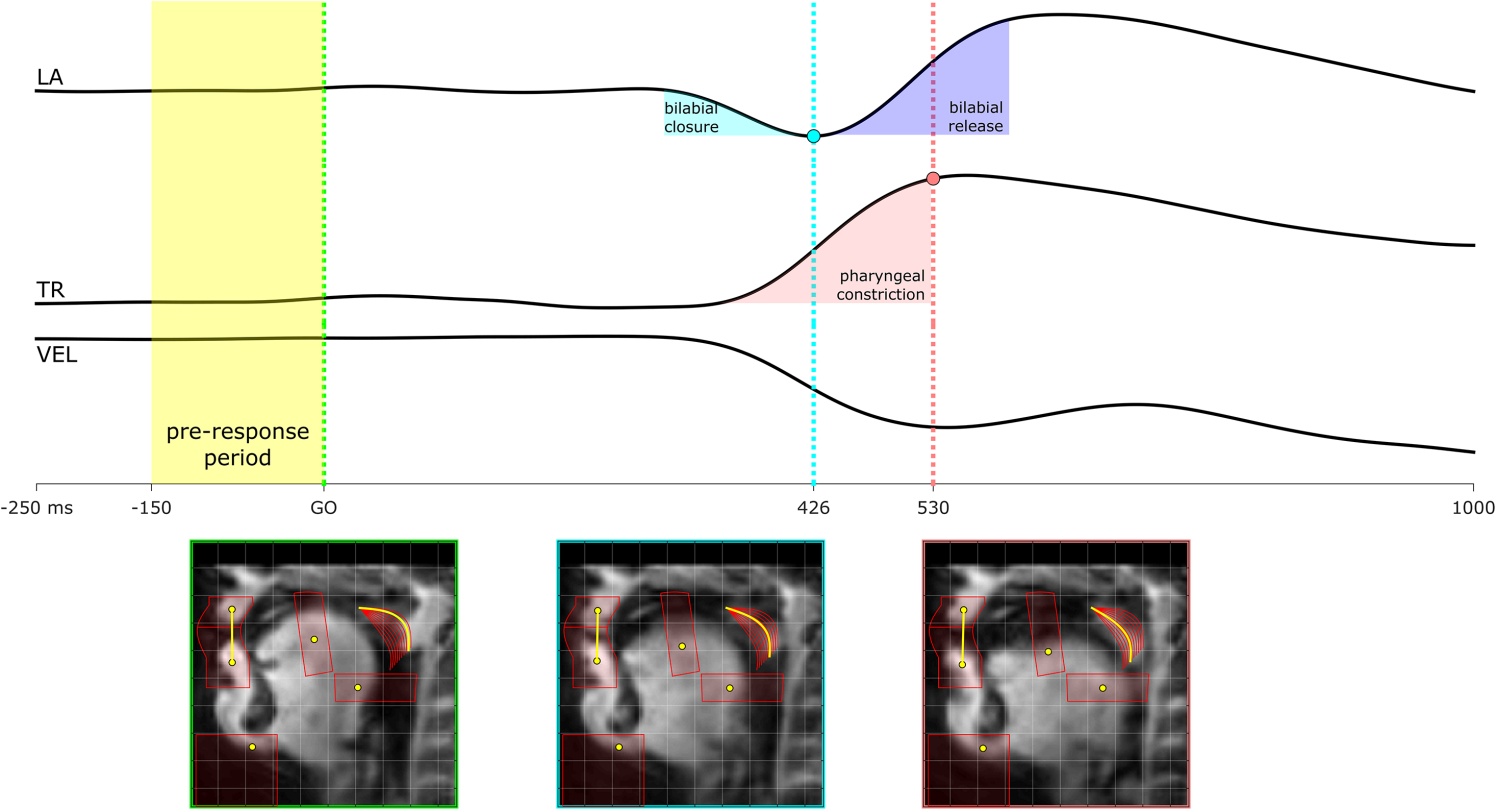
Example of articulatory feature time series: lip aperture (LA), tongue root horizontal position (TR), and velum position (VEL). The speaker produced "ma" in the constrained condition. The [m] involves a bilabial closure and velum opening, and the vowel involves a pharyngeal constriction with the tongue root.

An example of articulatory feature trajectories in shown in Fig 4, depicting a trial in the prepared, constrained condition with the response "ma". The [m] involves a bilabial closure, which is seen as a decrease in lip aperture (LA), and also velum opening, which is seen as a decrease in the velum (VEL) feature value. The vowel involves a pharyngeal constriction, which can be seen as a retraction of the tongue root (TR), along with a release of the bilabial closure (increase in LA). The pre-response period is defined as a 150 ms interval immediately preceding the go-signal.

### Data analysis

To identify effects of preparation on articulatory features, feature values were normalized within speakers to represent effects as a proportion of a typical range. For each subject and feature, the 5^th^ and 95^th^ percentiles of values were estimated from measures taken in a pre-response period, at vocalic target, and at consonantal target. Each feature was normalized by subtracting the 5^th^ percentile value and dividing by the range from 5^th^ to 95^th^ percentile. Occasional swallowing and respiration early in the pre-response interval result in extreme feature values and hence the percentile-based range provides a more appropriate normalization than the full range. Subsequently for ease of interpretation, the normalized values were re-centered within each constraint condition relative to the mean prepared condition targets. Specifically, JAW, LA, TT, and VEL feature values were re-centered to the prepared condition feature mean at consonantal target, and TR and TB feature values re-centered to prepared condition feature mean at vocalic target. For the LA and TT features in the unconstrained condition only, the consonantal target was substituted with value at release onset, because for many speakers a pre-response anticipatory closure precludes the identification of a point in time associated with consonantal target attainment.

The analyses focus on pre-response postures, which are considered here as the normalized articulatory feature values in a pre-response interval. The interval is defined as 150 ms preceding the go-signal. The duration of this interval was chosen to be fairly short in order to avoid conflating dynamic effects in pre-response posture with the pre-response posture immediately prior to the response signal (150 ms is approximately the typical duration of the tongue root movement from [i] to [a] in our data). For the TB, TR, and JAW features, two sample, two-sided t-tests were conducted within each combination of constraint condition and response place (labial, coronal). LA and TT features were only analyzed for labial and coronal responses, respectively. Kolmogorov-Smirnov tests showed that almost all of the oral feature samples were normally distributed (728/768, 94.8%); moreover the samples departing from normality did so only marginally (mean Kolmogorov-Smirnov statistic 0.23 with critical values around 0.185). However, departures from normality in the VEL feature samples were more prevalent and so effects in the VEL feature were tested by grouping responses according to nasality (+nasal, -nasal) and conducting Wilcoxon rank-sum tests. Subsequently a Benjamini-Hochberg procedure [35] was used with the p-values from all comparisons to adjust the significance threshold (adjusted p = 0.0080, α = 0.05). The counts of significant effects of preparation reported below are the number of these effects that remain significant after the adjustment.

## Results and Discussion

Comparisons of pre-response articulatory postures between prepared and unprepared responses supported the hypothesis that articulatory movements can be dissociated from linguistic units prior to the initiation of an utterance. Furthermore, a greater number of significant effects were observed in the unconstrained condition compared to the constrained condition, supporting the hypothesis that movement dissociations are facilitated in the absence of competing targets. Below we present the results and discuss their implications for models of speech production.

### Anticipatory posturing effects

**Fig 5:**
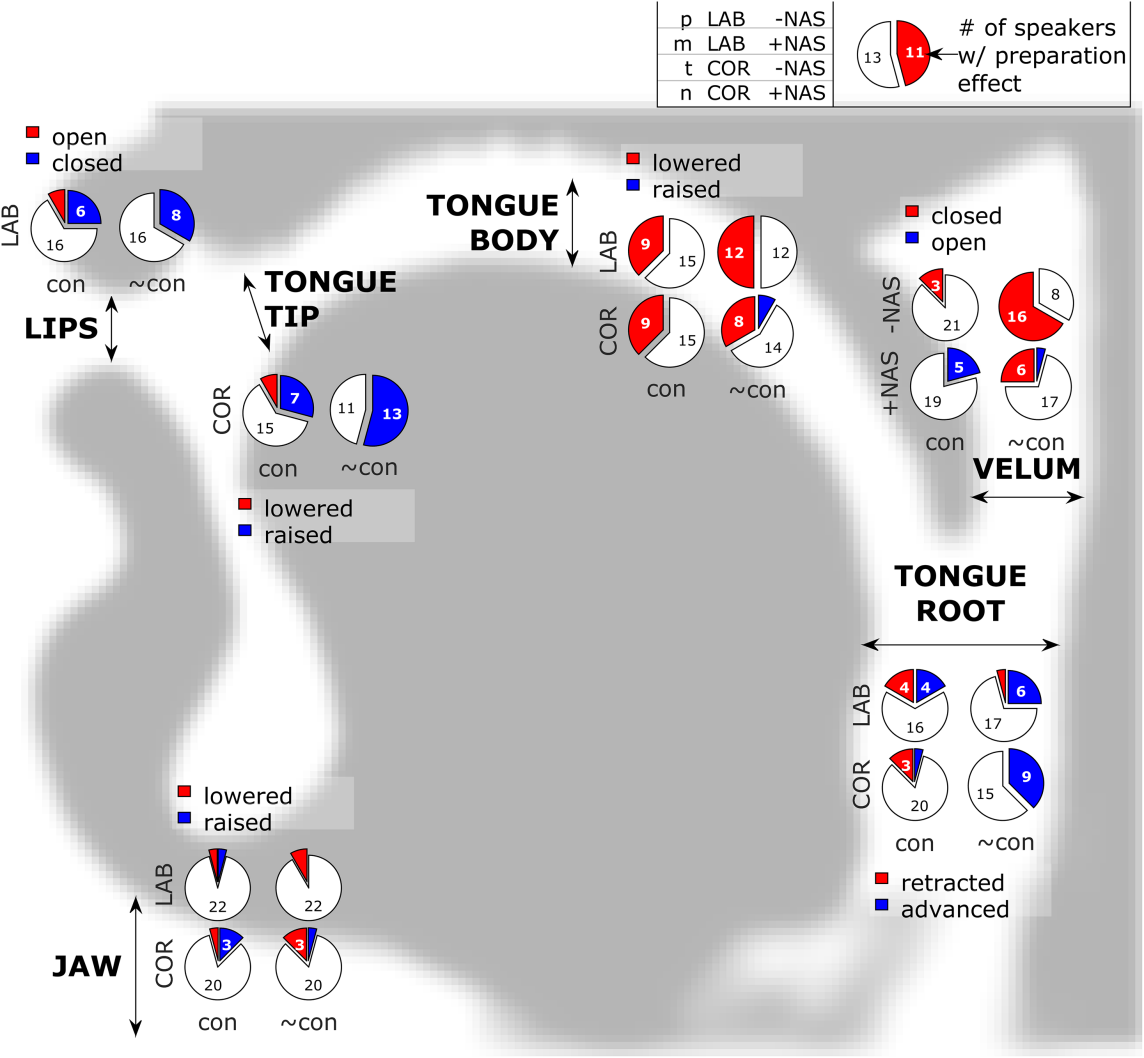
Summary of anticipatory posturing effects. Pie sections represent the number of participants showing a significant effect. Labial (lab: pa, ma) and coronal (cor: ta, na) responses are distinguished, as are nasal (+nas: ma, na) and non-nasal (-nas: pa, ta) responses.

**Fig 6:**
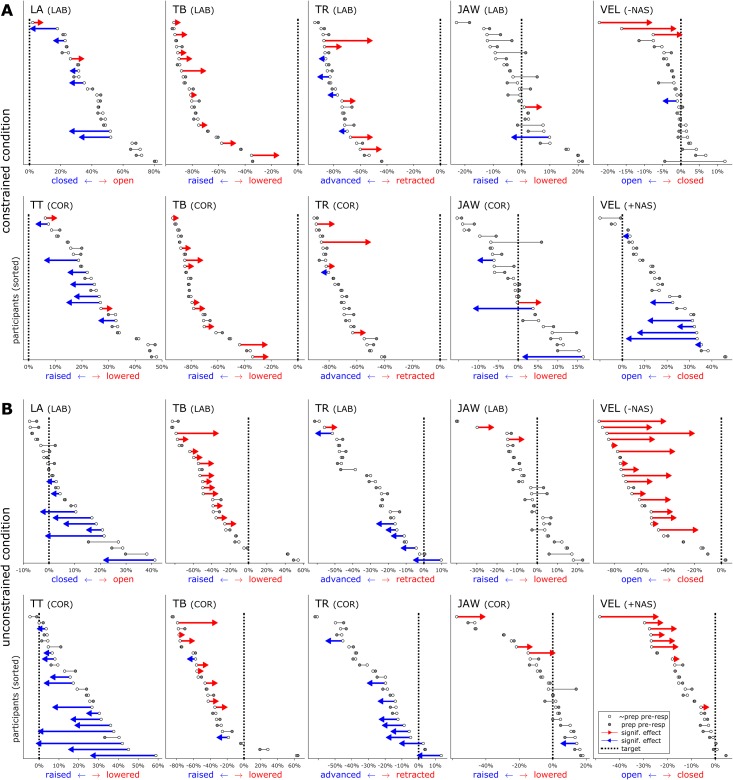
Effect directions and magnitudes for each speaker in each condition. Vertical lines show target position in prepared condition. Red/blue arrows indicate significant effects of preparation on pre-response posture.

Anticipatory postures were quantified with the time-varying articulatory features described in the preceding section. Fig 5 shows the number of speakers with significant anticipatory effects for each vocal tract feature in each constraint condition, Table 1 tabulates these effect counts, and Fig 6 shows effect magnitudes and directions for each articulatory feature in each response and constraint condition.

**Table 1:** Summary of anticipatory posturing effects.

	constrained		unconstrained	
Lips	closed^+C^	open	closed^+C^	open
LAB {p,m}	6	2	8	0
Jaw	raised	lowered	raised	lowered
LAB {p,m}	1	1	0	2
COR {t,n}	3	1	1	3
Tongue tip	raised^+C^	lowered	raised^+C^	lowered
COR {t,n}	7	2	13	0
Tongue body	raised	lowered^+V^	raised	lowered^+V^
LAB {p,m}	0	9	0	12
COR {t,n}	0	9	2	8
Tongue root	advanced^+C^	retracted^+V^	advanced^+C^	retracted^+V^
LAB {p,m}	4	4	6	1
COR {t,n}	1	3	9	0
Velum	open	closed^+C^	open	closed^+C^
-NAS {p,t}	0	3	0	16
	open^+C^	closed	open^+C^	closed
+NAS {m,n}	5	0	1	6

Counts represent the number of participants in each condition showing significant effects in each direction. (+C: effect direction corresponding to anticipation of consonantal target; +V: effect direction corresponding to anticipation of vocalic target). Labial (lab: pa, ma) and coronal (cor: ta, na) responses are distinguished, as are nasal (+nas: ma, na) and non-nasal (-nas: pa, ta) responses.

Most of the significant effects of preparation were assimilatory in nature, resulting in a posture that was more similar to an upcoming movement target. For example, anticipation of bilabial closure was observed for labial responses {p, m} in one-quarter and one-third of participants in the constrained and unconstrained conditions, respectively. More than half of the participants exhibited anticipatory raising of the tongue tip in unconstrained coronal responses {t, n}. Anticipatory lowering of the tongue body, which results in a posture closer to the vowel target, was observed in one-third to one-half of the participants across conditions and response categories. Because the position of the tongue body interacts strongly with tongue tip posture in coronals, the anticipatory lowering could be associated with either the consonantal or vocalic target. The fact that anticipatory lowering was also observed in labial responses supports the interpretation that tongue body lowering functions to anticipate the vocalic posture.

For some articulatory features there is ambiguity in the interpretation of anticipatory posturing. In the tongue root, in constrained labial responses, some participants exhibited anticipatory retraction, thereby anticipating the vocalic posture. Others exhibited anticipatory advancement of the tongue root, which may be a passive consequence of anticipatory raising of the jaw/lips for production of the consonant. However, an alternative interpretation is that the tongue root advancement arises from a dissimilatory effect: participants suppress the tongue root retraction plan as consequence of delaying response initiation, and consequently the tongue root posture is less similar to the vocalic posture than it is in unprepared responses. Note that a handful of dissimilatory effects were also observed in constrained responses with respect to lip aperture and tongue tip constriction: two participants exhibited a greater degree of pre-response lip aperture when the upcoming response was prepared; another two participants exhibited a similar effect involving tongue tip constriction.

The most frequently observed anticipatory posturing effect involved degree of velum opening in unconstrained, non-nasal responses: two-thirds of the participants closed the velum in anticipation of the upcoming non-nasal consonant. The frequency of this effect may be attributable to the nature of a generic speech-ready posture employed prior to the response in the unconstrained condition. As evident from comparing pre-response velum position in constrained vs. unconstrained responses (Fig 6A vs. 6B), speakers generally maintain the velum in a relatively open position when not constrained by any preceding speech targets. This accounts for why one-third of the participants also exhibited anticipatory closing in nasal responses: the target velum posture for {m, n} is more closed than the generic ready posture, although not as closed as it is for the non-nasals {p, t}. Anticipatory opening of the velum was also observed in constrained nasal responses, where the pre-response "ee" target requires a closed velum. This is likely related to anticipatory coarticulation of nasalization on a preceding vowel, a common phenomenon in English and many other languages.

Several significant effects on jaw elevation were observed, and these might be associated with consonantal or vocalic anticipation. The interpretation of these effects is complicated by the fact that jaw elevation participates synergistically with the lips and tongue for many movements; further complicating matters is the observation that about half of the participants exhibited pre-response postures with the jaw elevated relative to its position at the achievement of the consonantal target, while the other half exhibited a pre-response jaw position lower than the position at consonantal target (Fig 6, JAW).

In addition to showing that anticipatory movements are prevalent, the data show that these movements do not necessarily reach the target positions typically associated with a response. This can be readily seen in the unconstrained responses in Fig 6B by considering the displacement of pre-response anticipatory postures from their canonical targets (indicated by the dashed lines in each panel); specifically, examination of the LA, TT, and VEL postures shows that anticipatory postures do not always achieve the canonical target values associated with the response. These partial movements are of interest because current production models must make additional assumptions or incorporate new mechanisms in order to generate them.

Comparison of the frequency of anticipatory effects between constrained and unconstrained conditions provides some support for the hypothesis that movement dissociations are facilitated in the absence of a competing target. The difference was most pronounced in TT (7 vs. 13 effects) and TR (5 vs. 15 effects) features, and for non-nasal responses {p, t} in the VEL feature (3 vs. 16). In LA and TB features, the number of effects were similar. The interpretation of differences between constrained/unconstrained conditions in JAW and in VEL for nasal responses {m, n} is complicated for the reasons described above. It should be noted that the magnitudes of anticipatory posturing effects are not directly comparable between constraint conditions, because the unprepared response postures differ between these conditions. Nonetheless, the observed differences in the number of significant effects support the idea that pre-speech anticipatory posturing may be an extremal form of anticipatory movement that more prevalent in the absence of competition from other movement targets.

**Fig 7:**
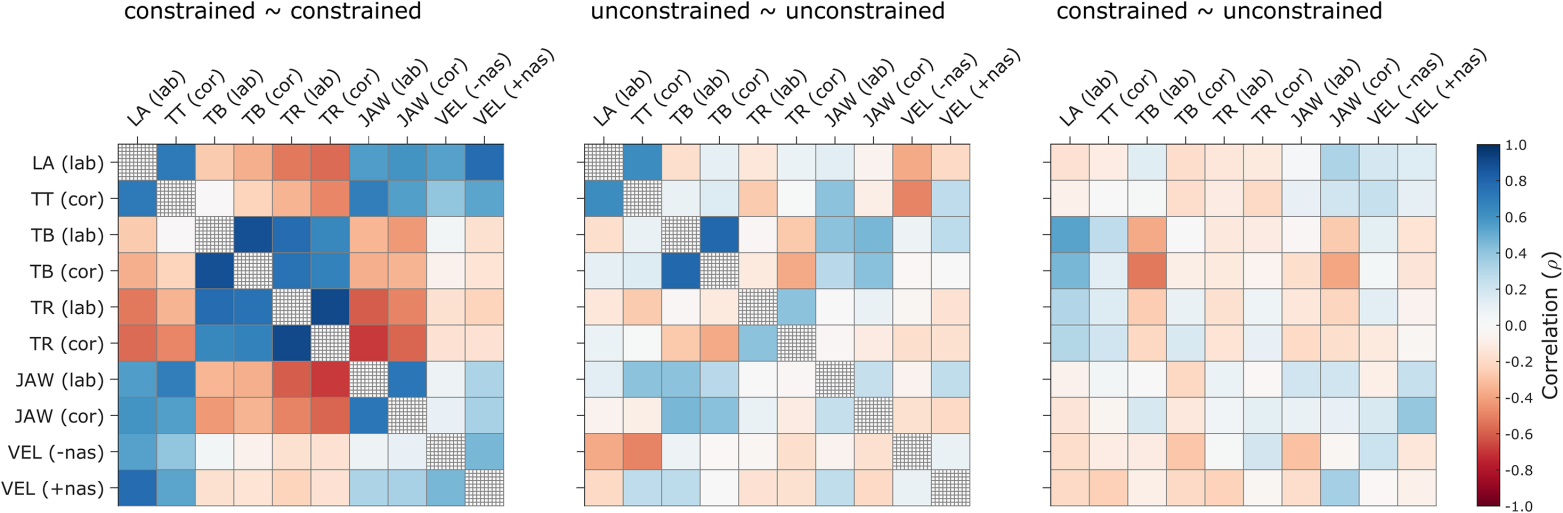
Across-participant correlations of anticipatory effects within and between constraint conditions. Correlations between labial and coronal responses, and nasal and non-nasal responses, are shown for each articulatory feature.

To assess the extent to which anticipatory posturing was response- and/or speaker-specific, by-speaker correlations were examined between anticipatory effects associated with each articulatory feature and response category, within and between constraint conditions, as shown in Fig 7 (values provided in S2 Table). The correlation magnitudes (absolute values) are of primary interest here, since the directions of anticipatory effects differ across features. The highest-magnitude anticipatory effect correlations were observed between coronal and labial responses within the tongue root and tongue body features, with *ρ* ≈ 0.90 in the constrained response condition. These high correlations suggest that anticipatory posturing of the dorsal and radial portions of the tongue function to anticipate the vocalic target, rather than being driven by anticipation of consonantal targets. Correlations of moderate magnitude (0.50 < |*ρ*| < 0.80) were observed between different features within and between response categories; these indicate that speakers who exhibited stronger anticipatory effects in one articulatory feature tended to exhibit stronger effects in other features. However, the observation that such correlations are merely moderate in magnitude, and are relatively weak in other feature combinations, indicates that anticipatory posturing is quite variable across speakers and depends on the response (i.e. differs between labial vs. coronal or nasal vs. non-nasal responses).

The extent to which anticipatory posturing effects are correlated between articulatory features also depends on the pre-response constraint condition. By distilling and averaging the between-category (labial vs. coronal) and within-category correlation magnitudes for each constraint condition, it was observed that average correlations are moderate in constrained responses: |*ρ*| = 0.51 (labial), 0.48 (coronal), 0.58 (labial-coronal), but weaker in unconstrained responses: |*ρ*| = 0.15 (labial), 0.20 (coronal), 0.28 (labial-coronal). Correlations between effects in constrained and unconstrained responses were likewise relatively weak (see S2 Table). The low correlation magnitudes observed in the unconstrained condition are likely a consequence of the greater flexibility speakers have for producing anticipatory postures in the absence of a preceding target posture.

## General Discussion

The above results support the hypotheses that articulatory movements can be dissociated from linguistic units and that such dissociations are facilitated in the absence of competing movement targets. How do these results relate to models of production in conversational speech? One concern that might be raised is whether non-word results necessarily extend to existing words. The consonant-vowel monosyllable responses examined in the current study involve articulatory patterns that are highly familiar to English speakers and frequently occur word-initially; in this regard the results should inform our understanding of conversational speech. Moreover, non-word monosyllables and disyllables are commonly used in articulatory studies and modeling [23,36,37], with the assumption that articulation of phonotactically regular sequences in non-words is not categorically different from articulation of those same sequences in familiar words. A potentially more worrying non-conversational aspect of the task is the restricted set of response options, which was deemed a necessary design feature in order to achieve sufficient statistical power. Tasks with a restricted set of response options may promote anticipatory behavior, and future studies should attempt to assess whether response-specific anticipatory posturing occurs in conversational speech.

The observed dissociations of articulatory movements from linguistic units may inform our understanding of the phenomenon of anticipatory coarticulation. Previous studies have demonstrated substantial inter-speaker variation in coarticulatory patterns [12,14]; the same form of variation was observed in pre-speech anticipatory posturing in the current study. Previous studies have also shown substantial cross-linguistic variation in coarticulation [13,15,38,39]; such variation may be related to language-specific differences in the role of articulatory tasks in systems of phonological contrast, and thus non-uniformity of anticipatory effects across articulatory features in the current study provides a basis for understanding how such differences might emerge.

An important question regarding the relation between pre-speech anticipation and coarticulation is whether pre-speech anticipatory movements are more closely related to long-distance coarticulation or to local coarticulation associated with overlap of adjacent gestures [40]. Long-distance coarticulatory patterns and their phonologization as harmonies have been argued to arise from different mechanisms than local coarticulation [41–43]. Of consonant harmonies, nasal and coronal harmonies are more common cross-linguistically than labial harmonies [42] and this was reflected in the greater prevalence of anticipatory effects in tongue tip, tongue body, and velum features than in the lip aperture feature. Moreover, pre-speech anticipatory posturing effects were observed minimally 300–500 ms prior to response initiation, which implies that pre-speech anticipatory movements occurred quite early relative to their associated targets. Taken together these results indicate that anticipatory movements dissociations are more closely associated with long-distance coarticulation than with local gestural overlap, and this entails that production models need two mechanisms for generating coarticulation: one that arises from temporally overlapping articulations, and one that arises from coactivation of motor plans.

A related consideration is that biomechanical optimization may be responsible for anticipatory movements and coarticulatory patterns. An optimization mechanism may serve to maximize mechanical advantage in a context-specific manner [24], in which case dissociations of movements from linguistic units could arise from parallel biomechanical and linguistic control processes. On the other hand, anticipatory movements could be more epiphenomenal, reflecting gesture-specific differences in inhibitory control of planned movements. Differentiating these possibilities is a high priority for advancing our understanding of speech motor control.

## Conclusion

The main finding of the current study is that individual speech movements associated with a given linguistic unit (i.e. segment or syllable) can be produced well in advance of other movements associated with the same unit. This observation is consistent with the hypothesis that individual speech movements can be selected and initiated independently of the linguistic units with which they are typically associated. Hence the selection and initiation of speech motor plans can occur independently of the selection and initiation of other motor plans associated with the same linguistic units. This finding is not consistent with models of speech production in which control over the timing of speech movements is necessarily organized by the selection of linguistics units such as segments and syllables. Instead, the results indicate that production models must incorporate an anticipatory mechanism that can induce anticipatory selection and movement execution independently from linguistically organized movement.

Also of importance is the finding that anticipatory movements exhibit substantial inter-speaker variation, particularly in relation to articulatory features and contextual factors. Speakers differ in the extent to which they produce anticipatory movements, they differ in which articulatory features they utilize for anticipatory posturing, and they differ in whether they exhibit anticipatory control of vocal tract posture when constrained by preceding articulatory targets. These findings reveal a new form of individual variation in speech production, and a better understanding of such variation is important for adducing constraints on speech acquisition and factors in the evolution of speech. The observations also raise the question of whether some clinical populations may exhibit abnormally high or low degrees of anticipatory posturing, and whether the behavior might be used as a diagnostic tool. Future studies should further investigate these effects and aim to determine the consequences of variation in anticipatory posturing for the subsequent production of speech.

## Supporting Information

S1 Table MRI scan and reconstruction parameters. For each subject, MRI scan parameters are shown: GE Scanner firmware version, number of spiral leaves, slice thickness (cm), tr (μs), te (μs), and sampling rate (Hz); and reconstruction parameters are shown: raw shift (μs), initial tolerance of TRACER algorithm, dynamic tolerance, and average iterations.
(DOCX)

S2 Table Effect correlations within and between constraint conditions. Articulatory feature pairs are shown on the left: tongue root (TR), tongue body (TB), tongue tip (TT), velum (VEL), lip aperture (LA); response target conditions are indicated in parentheses: coronal stops t, n (cor); labial stops p, m (lab); nasal stops m, n (+nas); oral stops p, t (-nas). Correlation coefficient (*ρ*) and p-value are shown for each correlation.
(DOCX)
