# Supplementary material for: Anticipatory Posturing of the Vocal Tract Reveals Dissociation of Speech Movement Plans from Linguistic Units
Source: PLoS One. 2016 Jan 13;11(1):e0146813. doi: 10.1371/journal.pone.0146813 (PMC4711920; doi:10.1371/journal.pone.0146813)
Supplement: S1 Table — For each subject, MRI scan parameters are shown: GE Scanner firmware version, number of spiral leaves, slice thickness (cm), tr (μs), te (μs), and sampling rate (Hz); and reconstruction parameters are shown: raw shift (μs), initial tolerance of TRACER algorithm, dynamic tolerance, and average iterations. (DOCX) [file pone.0146813.s001.docx]

**Table A.1. MRI scan and reconstruction parameters**

|  | MRI scan parameters | | | | | | reconstruction parameters | | | |
| --- | --- | --- | --- | --- | --- | --- | --- | --- | --- | --- |
| subj | firmware  version | number  of leaves | slice  thickness  (cm) | tr (μs) | te (μs) | sampling  rate (Hz) | raw  shift | initial  tolerance | dynamic  tolerance | average  iterations |
| S01 | 23HD4 | 36 | 2.5 | 5740 | 1188 | 174.2160 | -1 | 0.0125 | 0.0876 | 2.0444 |
| S02 | 23HD4 | 36 | 2.5 | 5740 | 1188 | 174.2160 | -1 | 0.0125 | 0.0781 | 2.1602 |
| S03 | 23HD4 | 24 | 2.5 | 6764 | 1188 | 147.8415 | -1 | 0.0125 | 0.0938 | 2.2778 |
| S04 | 23HD4 | 36 | 2.0 | 6296 | 1554 | 158.8310 | -1 | 0.0125 | 0.1250 | 2.0127 |
| S05 | 23HD4 | 36 | 2.0 | 6296 | 1554 | 158.8310 | -1 | 0.0125 | 0.0938 | 2.2350 |
| S06 | 23M4 | 36 | 2.0 | 4496 | 748 | 222.4199 | -1 | 0.0125 | 0.0703 | 2.2998 |
| S07 | 23M4 | 36 | 2.0 | 4496 | 748 | 222.4199 | -1 | 0.0125 | 0.0703 | 2.0856 |
| S08 | 23M4 | 36 | 2.0 | 4496 | 748 | 222.4199 | -1 | 0.0125 | 0.0703 | 2.1852 |
| S09 | 23M4 | 36 | 2.0 | 4496 | 748 | 222.4199 | 4 | 0.0050 | 0.0375 | 2.1377 |
| S10 | 23M4 | 36 | 2.0 | 4496 | 748 | 222.4199 | 4 | 0.0050 | 0.0375 | 2.1551 |
| S11 | 23M4 | 36 | 2.5 | 4328 | 632 | 231.0536 | 4 | 0.0050 | 0.0281 | 2.0565 |
| S12 | 23M4 | 36 | 2.5 | 4328 | 632 | 231.0536 | -1 | 0.0075 | 0.0750 | 2.2398 |
| S13 | 23M4 | 36 | 2.5 | 4328 | 632 | 231.0536 | -1 | 0.0075 | 0.0656 | 2.1222 |
| S14 | 23M4 | 36 | 2.5 | 4328 | 632 | 231.0536 | -1 | 0.0075 | 0.0656 | 2.0972 |
| S15 | 23M4 | 36 | 2.5 | 4328 | 632 | 231.0536 | -1 | 0.0075 | 0.0750 | 2.3028 |
| S16 | 23M4 | 36 | 2.5 | 4328 | 632 | 231.0536 | -1 | 0.0075 | 0.0656 | 2.0898 |
| S17 | 23M4 | 36 | 2.5 | 4328 | 632 | 231.0536 | -1 | 0.0075 | 0.0750 | 2.0824 |
| S18 | 24M4 | 36 | 2.5 | 4072 | 632 | 245.5796 | -1 | 0.0075 | 0.0938 | 2.2565 |
| S19 | 24M4 | 36 | 2.5 | 4072 | 632 | 245.5796 | -1 | 0.0125 | 0.1016 | 2.2870 |
| S20 | 24M4 | 36 | 2.5 | 4072 | 632 | 245.5796 | -1 | 0.0125 | 0.1016 | 2.0120 |
| S21 | 24M4 | 36 | 2.5 | 4072 | 632 | 245.5796 | -1 | 0.0125 | 0.1016 | 2.2352 |
| S22 | 24M4 | 36 | 2.5 | 4072 | 632 | 245.5796 | -1 | 0.0125 | 0.0938 | 2.3574 |
| S23 | 24M4 | 36 | 2.5 | 4072 | 632 | 245.5796 | -1 | 0.0125 | 0.0938 | 2.2880 |
| S24 | 24M4 | 36 | 2.5 | 4072 | 632 | 245.5796 | -1 | 0.0125 | 0.1094 | 2.1120 |
