# Supplementary material for: Anticipatory Posturing of the Vocal Tract Reveals Dissociation of Speech Movement Plans from Linguistic Units
Source: PLoS One. 2016 Jan 13;11(1):e0146813. doi: 10.1371/journal.pone.0146813 (PMC4711920; doi:10.1371/journal.pone.0146813)
Supplement: S2 Table — Articulatory feature pairs are shown on the left: tongue root (TR), tongue body (TB), tongue tip (TT), velum (VEL), lip aperture (LA); response target conditions are indicated in parentheses: coronal stops t, n (cor); labial stops p, m (lab); nasal stops m, n (+nas); oral stops p, t (-nas). Correlation coefficient (ρ) and p-value are shown for each correlation. (DOCX) [file pone.0146813.s002.docx]

**Table A.2. Effect correlations within and between constraint conditions**

| constrained ~ constrained | | | unconstrained ~ unconstrained | | |
| --- | --- | --- | --- | --- | --- |
|  | *ρ* | p-  value |  | *ρ* | p-  value |
| TR (cor) TR (lab) | 0.91 | <0.000 | TB (cor) TB (lab) | 0.79 | <0.000 |
| TB (cor) TB (lab) | 0.89 | <0.000 | TT (cor) LA (lab) | 0.63 | 0.001 |
| TR (lab) TB (lab) | 0.78 | <0.000 | JAW (cor) TB (lab) | 0.46 | 0.023 |
| VEL (+nas) LA (lab) | 0.77 | <0.000 | JAW (cor) TB (cor) | 0.43 | 0.038 |
| TR (lab) TB (cor) | 0.74 | <0.000 | JAW (lab) TT (cor) | 0.42 | 0.043 |
| JAW (cor) JAW (lab) | 0.73 | <0.000 | JAW (lab) TB (lab) | 0.42 | 0.043 |
| TT (cor) LA (lab) | 0.72 | <0.000 | TR (cor) TR (lab) | 0.41 | 0.044 |
| JAW (lab) TT (cor) | 0.69 | <0.000 | JAW (lab) TB (cor) | 0.29 | 0.176 |
| TR (cor) TB (cor) | 0.68 | <0.000 | VEL (+nas) TB (lab) | 0.28 | 0.193 |
| TR (cor) TB (lab) | 0.65 | 0.001 | VEL (+nas) TT (cor) | 0.27 | 0.205 |
| JAW (cor) LA (lab) | 0.60 | 0.002 | VEL (+nas) JAW (lab) | 0.26 | 0.214 |
| JAW (lab) LA (lab) | 0.56 | 0.004 | JAW (cor) JAW (lab) | 0.25 | 0.245 |
| JAW (cor) TT (cor) | 0.55 | 0.005 | TB (cor) TT (cor) | 0.15 | 0.486 |
| VEL (-nas) LA (lab) | 0.55 | 0.006 | JAW (lab) LA (lab) | 0.13 | 0.559 |
| VEL (+nas) TT (cor) | 0.53 | 0.008 | TB (cor) LA (lab) | 0.11 | 0.605 |
| VEL (+nas) VEL (-nas) | 0.46 | 0.022 | JAW (cor) TR (lab) | 0.10 | 0.642 |
| VEL (-nas) TT (cor) | 0.40 | 0.052 | TB (lab) TT (cor) | 0.10 | 0.643 |
| VEL (+nas) JAW (cor) | 0.34 | 0.104 | VEL (+nas) VEL (-nas) | 0.10 | 0.658 |
| VEL (+nas) JAW (lab) | 0.33 | 0.121 | TR (cor) LA (lab) | 0.09 | 0.677 |
| VEL (-nas) JAW (cor) | 0.10 | 0.628 | VEL (-nas) TB (lab) | 0.08 | 0.695 |
| VEL (-nas) JAW (lab) | 0.08 | 0.702 | TR (cor) TT (cor) | 0.02 | 0.927 |
| VEL (-nas) TB (lab) | 0.06 | 0.776 | VEL (+nas) TB (cor) | 0.02 | 0.944 |
| TB (lab) TT (cor) | -0.02 | 0.920 | JAW (lab) TR (lab) | 0.01 | 0.967 |
| VEL (-nas) TB (cor) | -0.08 | 0.727 | VEL (-nas) TB (cor) | -0.03 | 0.905 |
| VEL (+nas) TB (cor) | -0.15 | 0.479 | JAW (lab) TR (cor) | -0.04 | 0.868 |
| VEL (+nas) TR (cor) | -0.16 | 0.456 | TR (lab) TB (lab) | -0.04 | 0.862 |
| VEL (-nas) TR (cor) | -0.16 | 0.455 | VEL (-nas) TR (lab) | -0.04 | 0.842 |
| VEL (-nas) TR (lab) | -0.17 | 0.429 | JAW (cor) LA (lab) | -0.06 | 0.764 |
| VEL (+nas) TB (lab) | -0.17 | 0.426 | VEL (-nas) JAW (lab) | -0.07 | 0.762 |
| TB (cor) TT (cor) | -0.23 | 0.279 | JAW (cor) TT (cor) | -0.10 | 0.637 |
| VEL (+nas) TR (lab) | -0.23 | 0.277 | JAW (cor) TR (cor) | -0.11 | 0.602 |
| TB (lab) LA (lab) | -0.27 | 0.196 | TR (lab) TB (cor) | -0.12 | 0.574 |
| JAW (lab) TB (lab) | -0.34 | 0.105 | TR (lab) LA (lab) | -0.14 | 0.520 |
| TR (lab) TT (cor) | -0.35 | 0.098 | VEL (+nas) TR (lab) | -0.16 | 0.461 |
| JAW (cor) TB (cor) | -0.35 | 0.096 | VEL (-nas) JAW (cor) | -0.17 | 0.432 |
| JAW (lab) TB (cor) | -0.36 | 0.080 | VEL (+nas) TR (cor) | -0.17 | 0.423 |
| TB (cor) LA (lab) | -0.36 | 0.080 | TB (lab) LA (lab) | -0.18 | 0.412 |
| JAW (cor) TB (lab) | -0.43 | 0.035 | VEL (-nas) TR (cor) | -0.18 | 0.404 |
| TR (cor) TT (cor) | -0.49 | 0.016 | VEL (+nas) JAW (cor) | -0.20 | 0.337 |
| JAW (cor) TR (lab) | -0.50 | 0.013 | VEL (+nas) LA (lab) | -0.21 | 0.330 |
| TR (lab) LA (lab) | -0.53 | 0.008 | TR (lab) TT (cor) | -0.27 | 0.208 |
| TR (cor) LA (lab) | -0.57 | 0.004 | TR (cor) TB (lab) | -0.27 | 0.196 |
| JAW (cor) TR (cor) | -0.58 | 0.003 | VEL (-nas) LA (lab) | -0.38 | 0.070 |
| JAW (lab) TR (lab) | -0.60 | 0.002 | TR (cor) TB (cor) | -0.38 | 0.070 |
| JAW (lab) TR (cor) | -0.69 | <0.000 | VEL (-nas) TT (cor) | -0.50 | 0.013 |
| constrained ~ unconstrained | | | | | |
|  | *ρ* | p-  value |  | *ρ* | p-  value |
| LA (lab) TB (lab) | 0.54 | 0.007 | TB (lab) JAW (lab) | -0.02 | 0.943 |
| LA (lab) TB (cor) | 0.46 | 0.023 | VEL (-nas) VEL (+nas) | -0.03 | 0.902 |
| VEL (+nas) JAW (cor) | 0.40 | 0.055 | TR (cor) JAW (lab) | -0.03 | 0.898 |
| JAW (cor) VEL (+nas) | 0.35 | 0.098 | JAW (lab) TB (lab) | -0.04 | 0.858 |
| JAW (cor) LA (lab) | 0.33 | 0.121 | JAW (cor) VEL (-nas) | -0.04 | 0.838 |
| LA (lab) TR (lab) | 0.31 | 0.146 | VEL (+nas) TR (cor) | -0.04 | 0.835 |
| LA (lab) TR (cor) | 0.30 | 0.157 | TR (cor) VEL (+nas) | -0.05 | 0.830 |
| TT (cor) TB (lab) | 0.27 | 0.208 | TT (cor) JAW (cor) | -0.05 | 0.828 |
| VEL (+nas) JAW (lab) | 0.24 | 0.260 | TR (cor) TB (cor) | -0.06 | 0.792 |
| VEL (-nas) TT (cor) | 0.24 | 0.267 | VEL (+nas) TR (lab) | -0.07 | 0.746 |
| VEL (-nas) VEL (-nas) | 0.22 | 0.299 | LA (lab) JAW (lab) | -0.08 | 0.720 |
| JAW (cor) TT (cor) | 0.20 | 0.345 | LA (lab) TT (cor) | -0.08 | 0.716 |
| TT (cor) TR (cor) | 0.20 | 0.353 | VEL (+nas) VEL (+nas) | -0.08 | 0.694 |
| JAW (cor) JAW (lab) | 0.20 | 0.356 | VEL (-nas) JAW (lab) | -0.09 | 0.672 |
| JAW (lab) JAW (lab) | 0.20 | 0.360 | TB (lab) VEL (+nas) | -0.09 | 0.665 |
| TR (cor) VEL (-nas) | 0.19 | 0.362 | TB (cor) TB (cor) | -0.10 | 0.655 |
| VEL (-nas) LA (lab) | 0.18 | 0.392 | TT (cor) VEL (-nas) | -0.10 | 0.632 |
| TB (lab) JAW (cor) | 0.18 | 0.408 | TT (cor) LA (lab) | -0.11 | 0.624 |
| VEL (-nas) JAW (cor) | 0.17 | 0.425 | TB (lab) VEL (-nas) | -0.11 | 0.607 |
| TB (cor) TR (cor) | 0.17 | 0.437 | TR (cor) TB (lab) | -0.11 | 0.601 |
| TT (cor) TR (lab) | 0.15 | 0.472 | TR (lab) TT (cor) | -0.11 | 0.593 |
| VEL (+nas) LA (lab) | 0.14 | 0.502 | TR (cor) LA (lab) | -0.12 | 0.579 |
| TB (lab) LA (lab) | 0.14 | 0.526 | VEL (-nas) TR (cor) | -0.12 | 0.578 |
| VEL (-nas) TR (lab) | 0.13 | 0.549 | TR (lab) TB (cor) | -0.12 | 0.569 |
| TT (cor) TB (cor) | 0.13 | 0.560 | TR (lab) TB (lab) | -0.13 | 0.555 |
| VEL (-nas) TB (lab) | 0.12 | 0.561 | TB (cor) JAW (cor) | -0.13 | 0.545 |
| TR (cor) JAW (cor) | 0.12 | 0.563 | VEL (+nas) VEL (-nas) | -0.14 | 0.526 |
| VEL (+nas) TT (cor) | 0.11 | 0.597 | TR (lab) LA (lab) | -0.14 | 0.524 |
| JAW (lab) TT (cor) | 0.11 | 0.617 | JAW (lab) TR (lab) | -0.14 | 0.513 |
| JAW (cor) JAW (cor) | 0.11 | 0.617 | LA (lab) JAW (cor) | -0.14 | 0.502 |
| TR (cor) TR (cor) | 0.10 | 0.630 | VEL (+nas) TB (cor) | -0.15 | 0.494 |
| JAW (lab) JAW (cor) | 0.10 | 0.640 | TR (lab) TR (cor) | -0.15 | 0.480 |
| TB (cor) TR (lab) | 0.10 | 0.645 | VEL (+nas) TB (lab) | -0.15 | 0.478 |
| TR (lab) JAW (lab) | 0.09 | 0.684 | LA (lab) LA (lab) | -0.16 | 0.451 |
| TR (cor) TR (lab) | 0.07 | 0.742 | TR (lab) TR (lab) | -0.17 | 0.429 |
| TR (lab) JAW (cor) | 0.07 | 0.753 | JAW (lab) TB (cor) | -0.18 | 0.407 |
| TT (cor) JAW (lab) | 0.06 | 0.784 | JAW (cor) TR (cor) | -0.18 | 0.406 |
| VEL (-nas) TB (cor) | 0.04 | 0.841 | TB (cor) LA (lab) | -0.18 | 0.401 |
| JAW (lab) LA (lab) | 0.04 | 0.866 | LA (lab) VEL (-nas) | -0.18 | 0.397 |
| TB (lab) TT (cor) | 0.03 | 0.886 | TB (cor) TT (cor) | -0.19 | 0.382 |
| TR (lab) VEL (-nas) | 0.03 | 0.906 | JAW (lab) VEL (+nas) | -0.19 | 0.382 |
| TT (cor) TT (cor) | 0.01 | 0.952 | JAW (lab) TR (cor) | -0.20 | 0.358 |
| TB (cor) TB (lab) | 0.01 | 0.974 | TR (cor) TT (cor) | -0.21 | 0.331 |
|  |  |  | LA (lab) VEL (+nas) | -0.21 | 0.327 |
|  |  |  | TB (cor) VEL (+nas) | -0.21 | 0.324 |
|  |  |  | TB (lab) TR (cor) | -0.21 | 0.315 |
|  |  |  | TB (cor) JAW (lab) | -0.22 | 0.305 |
|  |  |  | JAW (cor) TR (lab) | -0.23 | 0.288 |
|  |  |  | TR (lab) VEL (+nas) | -0.24 | 0.264 |
|  |  |  | TT (cor) VEL (+nas) | -0.24 | 0.252 |
|  |  |  | TB (lab) TR (lab) | -0.26 | 0.214 |
|  |  |  | JAW (cor) TB (lab) | -0.27 | 0.205 |
|  |  |  | TB (cor) VEL (-nas) | -0.29 | 0.173 |
|  |  |  | JAW (lab) VEL (-nas) | -0.30 | 0.157 |
|  |  |  | TB (lab) TB (lab) | -0.38 | 0.067 |
|  |  |  | JAW (cor) TB (cor) | -0.39 | 0.059 |
|  |  |  | TB (lab) TB (cor) | -0.52 | 0.009 |
